# Supplementary material for: Accurate Diagnosis of High-Risk Pulmonary Nodules Using a Non-Invasive Epigenetic Biomarker Test
Source: Cancers (Basel). 2025 Mar 7;17(6):916. doi: 10.3390/cancers17060916 (PMC11940740; doi:10.3390/cancers17060916)
Supplement: Supplementary file 1 [file cancers-17-00916-s001.zip › Supplementary_Table_0220.1 .pdf]

Supplementary Table S1. Five Quantitative Epigenetic Features

No.	Abbreviation	Full name
1	H3.1	Nucleosome containing H3.1 variant (H3.1-nucleosome)
2	H3K9Ac	Nucleosome containing Histone H3 acetyl Lys9 (H3K9Ac-nucleosome)
3	H3K9Me3	Nucleosome containing Histone 3 lysine 9 trimethylation (H3K9Me3-nucleosome)
4	H3K27Me3	Nucleosome containing Histone 3 lysine 27 trimethylation (H3K27Me3-nucleosome)
5	H3K36Me3	Nucleosome containing Histone 3 lysine 36 trimethylation (H3K36Me3-nucleosome)

Supplementary Table S2. Summary of Epigenetic Marker Levels in Blood

		**Average (ng/mL)**	**Median (ng/mL)**	**Standard Deviation**	**Min**	**Max**
H3.1	nonCancer	26.1	16.4	54.2	2.5	509.5
	Cancer	34.0	26.1	39.3	1.9	518.0
H3K27Me3	nonCancer	16.6	11.7	25.1	3.8	261.7
	Cancer	17.8	14.6	16.3	2.7	252.3
H3K9Ac	nonCancer	4.9	4.3	4.5	1.9	57.4
H3K9Ac	Cancer	4.5	4.3	1.5	0.9	27.4
H3K9Me3	nonCancer	17.4	13.1	22.5	4.8	246.0
H3K9Me3	Cancer	17.8	15.1	12.5	4.3	187.0
H3K36Me3	nonCancer	9.7	7.5	18.1	4.1	227.3
H3K36Me3	Cancer	9.5	8.2	6.6	4.0	88.5

Supplementary Table S3. Figure 2 AUC Comparison

Comparison	AUC 1	AUC 2	Difference in AUC (D)	p.value*
Training vs Validation dataset	0.740	0.858	-2.37030	0.019
Training vs Test dataset	0.740	0.789	-0.94108	0.347
Validation vs Test dataset	0.858	0.789	1.15320	0.250

*In all cases, difference (D) in AUC and p.value was calculated using roc.test() function from pROC package. Bootstrap test was used, resampling the data 2000 times (boot.n = 2000) preserving the proportion of classes (boot.stratified = 1)

Supplementary Table S4. Figure 3 AUC Comparison

Comparison	*AUC 1*	*AUC 2*	*Difference in AUC (D)*	*p.value**
Mayo vs VA model	*0.570*	*0.503*	*0.950*	*0.342*
Mayo vs EB model	*0.570*	*0.858*	*-4.011*	*6.04e-05*
VA vs EB model	*0.503*	*0.858*	*-5.089*	*7.82e-07*

*In all cases, difference (D) in AUC and p.value was calculated using roc.test() function from pROC package. Bootstrap test was used, resampling the data 2000 times (boot.n = 2000) preserving the proportion of classes (boot.stratified = 1)
