# Supplementary material for: Accurate Diagnosis of High-Risk Pulmonary Nodules Using a Non-Invasive Epigenetic Biomarker Test
Source: Cancers (Basel). 2025 Mar 7;17(6):916. doi: 10.3390/cancers17060916 (PMC11940740; doi:10.3390/cancers17060916)
Supplement: Supplementary file 1 [file cancers-17-00916-s001.zip › Supplenmentary R code .pdf]

```
#####################################
######### Setup Data ###############
#####################################

# Required packages
library(readxl)
library(tidyverse)
library(ggplot2)
library(pROC)
library(tidymodels)
library(caret)

# Read data
alldata_my <- read_excel("./alldata.xlsx")
trainData <- read_excel("./trainData.xlsx")
validationData <- read_excel("./validationData.xlsx")
validationData <- validationData[,1:10]

# Change name from ground glass opacity to GGO
alldata_my$Image <- gsub("ground glass opacity", "GGO", alldata_my$Image,
fixed=TRUE)

#########################################
### Create Train, Validation & Test ###
#########################################

# Convert 0 == nonCancer and 1 == Cancer
alldata_my$cancer <- gsub("0", "nonCancer", alldata_my$cancer, fixed=TRUE)
alldata_my$cancer <- gsub("1", "Cancer", alldata_my$cancer, fixed=TRUE)

# Split into training, validation, and test sets
set.seed(20)
train_val_size <- floor(0.75 * nrow(alldata_my))
train_val_Indices <- sample(seq_len(nrow(alldata_my)), size = train_val_size)

train_val_data <- alldata_my[train_val_Indices, ]
test_data <- alldata_my[-train_val_Indices, ]
```

```
set.seed(123)
train_size <- floor(0.8 * nrow(train_val_data))
train_Indices <- sample(seq_len(nrow(train_val_data)), size = train_size)

train_data <- train_val_data[train_Indices, ]
val_data <- train_val_data[-train_Indices, ]

########################################
### Logistic Regression Model #########
########################################

# Create factor levels
train_data$cancer <- factor(train_data$cancer, levels = c("nonCancer","Cancer"))

# Fit logistic model
logisticModel <- glm(cancer ~ nH3.1 + nH3K27Me3, family = binomial, data =
train_data)
summary(logisticModel)

########################################
### Predictions & ROC Analysis ########
########################################

val_data$predicted_prob <- predict(logisticModel, newdata = val_data,
type="response")
roc_curve_val <- roc(val_data$cancer, val_data$predicted_prob, levels =
c("nonCancer", "Cancer"))
auc(roc_curve_val)

plot(roc_curve_val, col="blue", main="Validation Data", print.auc=TRUE,
print.auc.col="blue")

# Determine optimal threshold
a <- data.frame(thres=roc_curve_val$thresholds,
                sensitivities=roc_curve_val$sensitivities,
                YOUDEN=(roc_curve_val$sensitivities +
roc_curve_val$specificities - 1))
```

```
optimal_threshold <- a %>% filter(sensitivities > 0.8) %>% arrange(-YOUDEN) %>%
slice(1)
threshold_val <- optimal_threshold$thres

#########################################
### Confusion Matrix ##################
#########################################

val_data$predicted_class <- as.factor(ifelse(val_data$predicted_prob >
threshold_val, "Cancer", "nonCancer"))
confusionMatrix(val_data$predicted_class, val_data$cancer)

#########################################
### Test Data Predictions ##############
#########################################

test_data$predicted_prob <- predict(logisticModel, newdata = test_data,
type="response")
roc_test <- roc(test_data$cancer, test_data$predicted_prob, levels = c("nonCancer",
"Cancer"))
auc(roc_test)

plot(roc_test, col="red", main="Test Data", print.auc=TRUE, print.auc.col="red")

test_data$predicted_class <- as.factor(ifelse(test_data$predicted_prob >
threshold_val, "Cancer", "nonCancer"))
confusionMatrix(test_data$predicted_class, test_data$cancer)

#########################################
### Subgroup Analysis: RADS & SPG #####
#########################################

# Define RADS subgroups
testdata_RADS4 <- test_data %>% filter(RADS %in% c("4A", "4B", "4X"))

testdata_Solid <- test_data %>% filter(Image == "Solid")
testdata_Partsolid <- test_data %>% filter(Image == "Part-Solid")
testdata_GGO <- test_data %>% filter(Image == "GGO")
```

```
# Function for confusion matrix per subgroup
analyze_subgroup <- function(subgroup_data, label) {
   predicted_class <- as.factor(ifelse(subgroup_data$predicted_prob > threshold_val,
"Cancer", "nonCancer"))
   print(paste("Confusion Matrix for", label))
   print(confusionMatrix(predicted_class, subgroup_data$cancer))
}

# Run analysis
analyze_subgroup(testdata_RADS4, "RADS 4")
analyze_subgroup(testdata_Solid, "Solid")
analyze_subgroup(testdata_Partsolid, "Part-Solid")
analyze_subgroup(testdata_GGO, "GGO")
```
